# Local Pedicled Flaps and Biological Implant Options for Patients Undergoing Proctectomy for Crohn’s Disease When an Omental Pedicle Flap Is Not Possible

**DOI:** 10.3390/medicina61071153

**Published:** 2025-06-26

**Authors:** Jacob Baxter, Ian S. Reynolds, Nho V. Tran, David W. Larson, Kellie L. Mathis, Nicholas P. McKenna

**Affiliations:** 1 Division of Colon and Rectal Surgery, Mayo Clinic, Rochester, MN 55905, USA; baxter.jacob@mayo.edu (J.B.); ianreynolds@rcsi.com (I.S.R.); larson.david2@mayo.edu (D.W.L.); mathis.kellie@mayo.edu (K.L.M.); 2 Division of Plastic Surgery, Mayo Clinic, Rochester, MN 55905, USA; tran.nho@mayo.edu

**Keywords:** Crohn’s disease, proctectomy, perineal wound, pedicled flaps

## Abstract

*Background and Objectives:* Perineal wound complications and pelvic fluid collections or abscesses following proctectomy for Crohn’s disease are a common cause of morbidity and might be mitigated by filling the pelvis and occluding the pelvic inlet with a flap. Alternative flap options can be considered when inadequate omentum is available and when avoiding myofasciocutaneous flaps. *Materials and Methods:* A retrospective review of our Crohn’s proctectomy database was conducted to identify patients who underwent a non-omental or non-myofasciocutaneous local pedicle flap to their pelvis or pelvic exclusion using biological material during surgery. The techniques and outcomes of these alternative techniques are described in detail. *Results:* 228 patients underwent proctectomy for Crohn’s disease during the 10-year study period. However, only six patients had their pelvis filled or pelvic inlet occluded using a non-omental, non-myofasciocutaneous local pedicled flap or biological material. The techniques identified included two sigmoid mesocolic flaps, one peritoneal, preperitoneal fat and falciform ligament flap, one perivesical fat flap, one Gerota’s fat pad flap, and one bovine pericardial patch assisted pelvic exclusion. These flaps’ clinicopathological and operative characteristics, surgical outcomes, and technical aspects are described. *Conclusions:* When greater omentum is unavailable or inadequate and myofasciocutaneous flaps need to be avoided, local pedicled flaps using a range of intra-abdominal tissues or biological material can be used to fill the pelvis or occlude the pelvic inlet after proctectomy for Crohn’s disease. These techniques may help to prevent short and long-term complications associated with having a pelvic dead space.

## 1. Introduction

The impact of the empty pelvis has been clearly defined in the setting of pelvic exenteration. However, the significance of the pelvic dead space after extirpation of the rectum in the setting of Crohn’s disease (CD) remains somewhat unknown [1,2,3]. Empty pelvis syndrome (EPS) is a term that is used most frequently in the setting of patients’ post pelvic exenteration and encompasses several potential complications following pelvic organ resection, including bowel obstruction, wound breakdown, pelvic abscess, perineal hernia, and fistulae. Patients with CD may be at higher risk for these EPS-related complications due to their underlying inflammatory bowel disease. Chronic perineal wounds following proctectomy for CD represent a common and debilitating source of morbidity for patients [4]. The underlying cause of these wounds is multifactorial and includes patient factors such as nutritional status, use of immunosuppressant medications, and disease phenotype and severity. We believe that filling the pelvic void and occluding the pelvic inlet may prevent the development of EPS and reduce the risk of complications. How to do this remains challenging and without a widely accepted standard of care. Numerous methods have been employed, including omental pedicle flaps (OPF) or omental slips, myofasciocutaneous flaps, mesh, and other prosthetic devices [5,6].

Using native tissue flaps to fill the pelvic dead space decreases the risk of pelvic sterile fluid collections, pelvic abscesses, wound dehiscence, chronic perineal sinus or fistula formation, and perineal hernia development. Furthermore, reducing the pelvic dead space is believed to reduce the risk of future small bowel obstructions by preventing bowel loops from adhering to raw surfaces in the recently operated pelvis. The vertical rectus abdominus myocutaneous flap (VRAM) is a standard flap used for perineal reconstruction. While this is a good option in many patients for the prevention of short and long-term pelvic complications after proctectomy for CD, it does introduce potential problems, including immediate vascular complications requiring urgent reoperation and longer-term complications such as abdominal wall hernia formation [7]. Furthermore, complex pedicled and free flap reconstructions necessitate the involvement of a plastic surgery team, which may constrain OR availability and increase cost. We have previously published our outcomes regarding the use of OPF in the setting of CD [5]. In certain circumstances, an OPF is unavailable due to diminutive or absent greater omentum secondary to malnutrition, low BMI, or previous surgery. In these circumstances, alternative options can be considered before resorting to myofasciocutaneous flaps.

This study aims to highlight alternative flap options in patients who lack adequate greater omentum to fill the pelvis and occlude the pelvic inlet to prevent pelvic and perineal complications after proctectomy for CD.

## 2. Materials and Methods

Institutional review board approval was granted for this study (IRB#: 18000-190). The study was conducted following the Declaration of Helsinki. Informed consent was obtained from all patients involved in this study. A retrospective review of a prospectively maintained CD proctectomy database was performed. The database included details of all proctectomies performed for CD in our institution in the 10 years between 1 January 2014 and 31 December 2023. Patients who underwent proctectomy for complications of perianal Crohn’s disease or Crohn’s proctitis were eligible for inclusion. Patients included in the study underwent primary perineal closure (PPC) with a native pedicled tissue flap or biological implant. Patients who had an OPF or myofasciocutaneous flap were excluded from the study. The following clinical and surgical technique variables were extracted for each patient: age, sex, smoking status, diabetes, body mass index (BMI), use of immunosuppressants within six weeks of surgery, presence of perianal fistula(e), previous resection, procedure, approach (open, perineal, laparoscopic, robotic, hybrid), type of flap, type of perineal dissection (intersphincteric or extrasphincteric), type of suture used for perineal skin closure, use of incisional wound VAC, pelvic drain placement, operative time, and blood loss. The following pathological variables were extracted for each patient: sharply demarcated segment involvement, linear ulcerations, skip areas, continuous involvement, fat creeping, macroscopic evidence of fissures or fistulae, stricture, marked mucosal atrophy, transmural inflammation, fissuring ulceration, non-necrotizing granulomata, lymphoid aggregates in all layers of the bowel wall, dense mucosal inflammation, crypt abscesses, thickening of muscularis mucosa, proximal margin involvement, and distal margin involvement. Unplanned returns to the OR for perineal wound complications, the need for radiologic-guided drainage of pelvic abscesses, length of stay (LOS), unplanned re-admissions, small bowel obstructions, and perineal hernia formation were documented for all patients.

**Figure 1:**
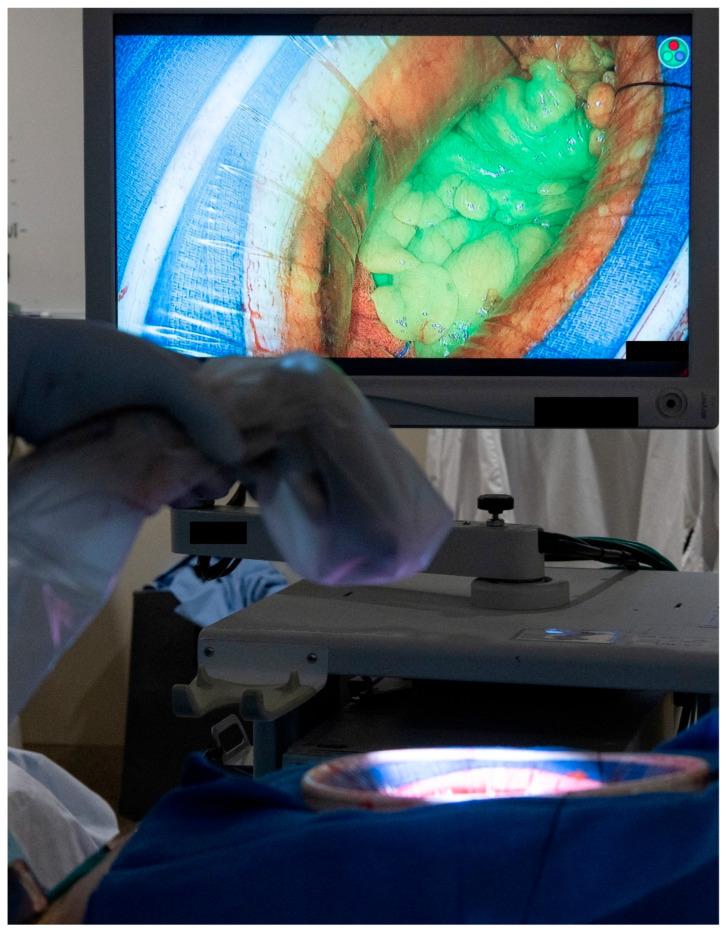
Intra-operative assessment of tissue perfusion using the indocyanine green and the SPY-PHI portable handheld imaging system from Stryker. (Used with permission from the Mayo Foundation for Medical Education and Research; all rights reserved).

Each patient’s clinicopathological and operative characteristics are described in the Section 3, along with a technical description of each flap type and its associated outcomes. Continuous variables are expressed as medians with interquartile range (IQR). Adequate blood supply to each of the flaps was confirmed using either Doppler ultrasonography or the use of indocyanine green. The protocol in our institution is to administer 3 mL of indocyanine green followed by an immediate 5 mL flush of normal saline. The tissue of interest is then immediately assessed for adequate perfusion using Stryker’s SPY-PHI portable handheld imaging system (See Figure 1).

## 3. Results

Two hundred twenty-eight patients underwent proctectomy for CD during the ten-year study period. Two hundred twenty-two patients were ineligible due to no flap (*n* = 148), an omental pedicle flap (*n* = 71), or a VRAM (*n* = 3), thus leaving six patients who underwent PPC with another type of local pedicled flap or had exclusion of their pelvis using a biological implant. In these six patients, a sigmoid mesocolic flap was used in two patients, a peritoneal, preperitoneal fat, and falciform ligament flap was used in one patient, a perivesical fat flap was used in one patient, a Gerota’s fat pad flap in one patient, and a bovine pericardial patch was used in the final patient. Several flaps required assistance from a plastic surgeon to mobilize the flap and ensure that the flap’s perfusion was not compromised. The cohort included four females and two males. The indication for surgery was complex and refractory fistulizing perianal disease in three patients, a rectovaginal fistula in one patient, a rectovaginal fistula and anal stenosis in one patient, and high-grade dysplasia of the rectum with Crohn’s proctitis in the final patient. The perineal skin was closed with interrupted nylon sutures in five patients and a subcuticular monocryl suture in the remaining patient. Clinical and operative data for the six patients are highlighted in Table 1, and pathological data in Table 2. All operations were performed in an open fashion. The median operating time was 471 min (IQR = 238 min). The median blood loss was 500 mL (IQR = 828 mL). The median follow-up time was 26 months (IQR = 36 months). No patients required a return to the operating room for complications related to the flap or their perineal wound, and there was only one unplanned re-admission to the hospital. The patient with the perivesical fat flap was re-admitted to the hospital with a 4.7 × 4.0 cm pelvic abscess not adequately controlled by their surgical drain. They had a second pelvic drain inserted by interventional radiology, which remained in place for two months until complete resolution of the pelvic collection was confirmed on repeat imaging. There were no prolonged perineal wound issues in any of the patients. No postoperative small bowel obstructions or perineal hernias have been identified in this cohort of patients to date.

### 3.1. Sigmoid Mesocolic Flaps

**Figure 2:**
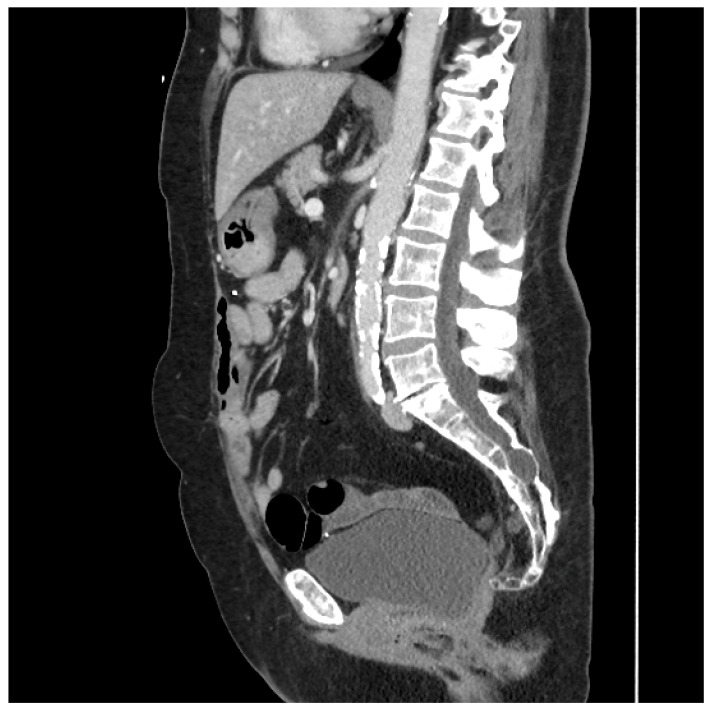
Sagittal CT scan performed four years after proctocolectomy and sigmoid mesocolic flap. The mesocolic flap is seen here filling the pelvis and adherent to the back of the bladder, forming a sling-like configuration, thus preventing the small bowel from adhering to the proctectomy resection bed in the pelvis. (Used with permission from the Mayo Foundation for Medical Education and Research; all rights reserved).

This type of flap was used in two patients. The first underwent total proctocolectomy for fistulizing perianal disease and a rectovaginal fistula. She had a diminutive greater omentum found during surgery that was deemed unsuitable for an omental pedicle flap. Close colonic and rectal dissection were carried out, which left a sufficient amount of sigmoid mesocolon in situ. The sigmoid mesocolon was advanced inferiorly and sutured to the pelvic brim to close it off and prevent the small bowel from entering the pelvis. The blood supply of the mesocolon was preserved as the inferior mesenteric artery was not ligated, and the superior rectal artery was left intact. A 15 Fr drain was left in the pelvis at the end of the operation. A CT scan performed four years after surgery demonstrated the resilience of this flap and its prolonged ability to prevent the small bowel from adhering to the proctectomy resection bed (See Figure 2).

The second patient underwent a completion proctocolectomy for endoscopically unresectable dysplasia of the rectum in the setting of long-standing CD. This patient had no remaining greater omentum, which was resected during a previous subtotal colectomy. He underwent close colon and rectal dissection, leaving a significant volume of sigmoid mesocolon behind. Frozen section analysis found only low-grade dysplasia in the rectum. Thus, total mesorectal excision was deemed unnecessary. The sigmoid mesentery was mobilized off the retroperitoneum and advanced inferiorly to occlude the pelvic inlet. The bladder was then mobilized and dropped inferiorly into the pelvis. 3-0 vicryl sutures were used to secure the posterior bladder to the sigmoid mesocolic flap and further occlude the pelvis. A 19 Fr drain was placed posterior to the flap with the tip deep in the pelvis.

### 3.2. Peritoneal, Preperitoneal Fat and Falciform Ligament Flap

**Figure 3:**
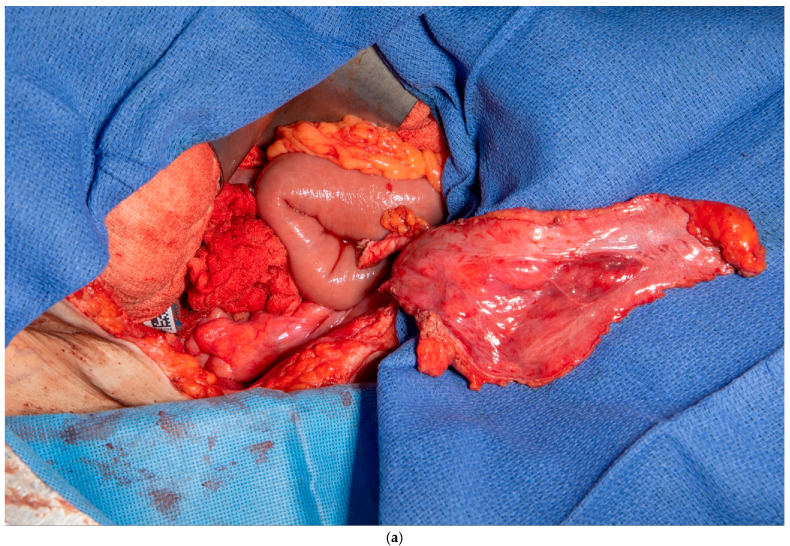

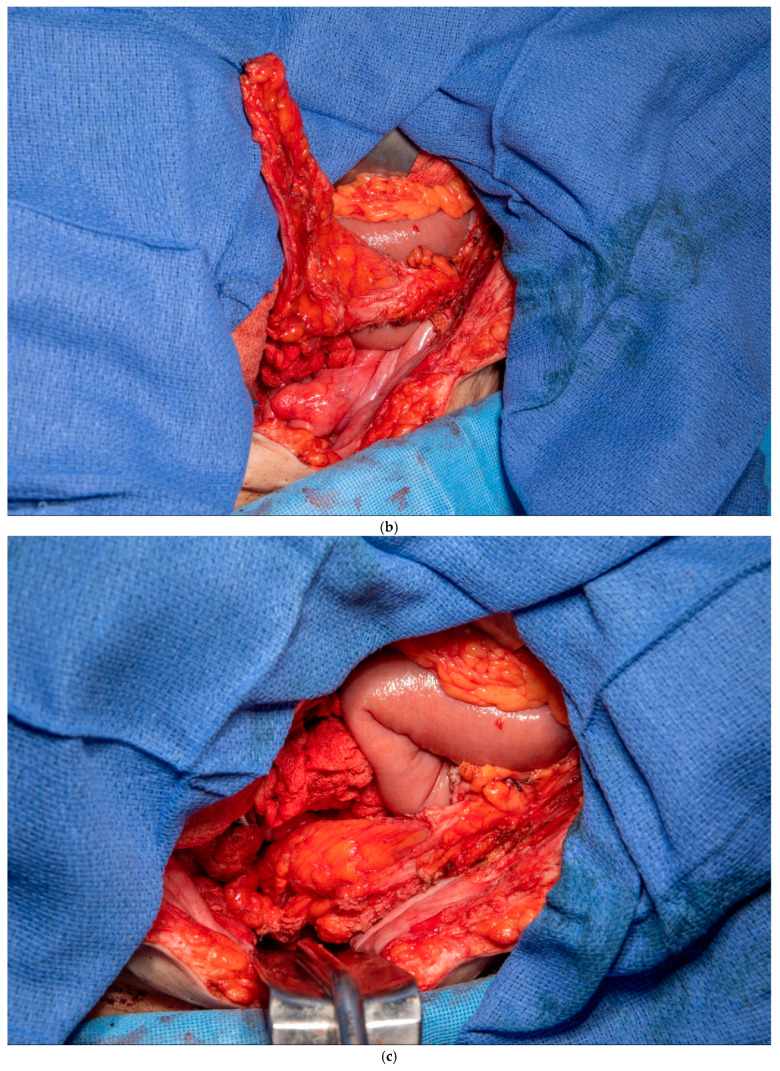
(**a**) Peritoneum and preperitoneal fat dissected off the patient’s left posterior sheath. (Used with permission from the Mayo Foundation for Medical Education and Research; all rights reserved). (**b**) Peritoneum, preperitoneal fat, and falciform ligament dissected off the patient’s right posterior sheath. (Used with permission from the Mayo Foundation for Medical Education and Research; all rights reserved). (**c**) Bilateral pedicled flaps sutured to the pelvic inlet, creating a sling to prevent a small bowel from falling into the pelvis. (Used with permission from the Mayo Foundation for Medical Education and Research; all rights reserved).

This patient had no greater omentum left, as it was removed during a previous total abdominal colectomy. A VRAM was also not possible because she had bilateral rectus abdominus divisions from transverse incisions as a child, and no perforators to the skin could be identified. However, she had some remaining deep perforating branches of the inferior epigastric artery. She required a proctectomy because of a rectovaginal fistula and anal stenosis. Once the midline laparotomy incision was made with the assistance of a plastic surgeon, the peritoneum and preperitoneal fat were dissected off her posterior sheath bilaterally (See Figure 3a,b). The falciform ligament was ligated, divided as high as possible, and kept in continuity with the peritoneum and peritoneal fat flap. A persistent branch of the deep inferior epigastric vessel supplying this tissue was identified and protected. The peritoneum was also mobilized off the lower aspect of the rectus muscle to allow the flap to be advanced toward the pelvis. Doppler ultrasonography was used to confirm that blood supply to the flap was maintained. The flap was then secured to the pelvic inlet in a sling-like fashion to keep the small intestine out of the pelvis (See Figure 3c). A 19 Fr drain was placed in the pelvis at the end of the case.

### 3.3. Perivesical Fat Flap

**Figure 4:**
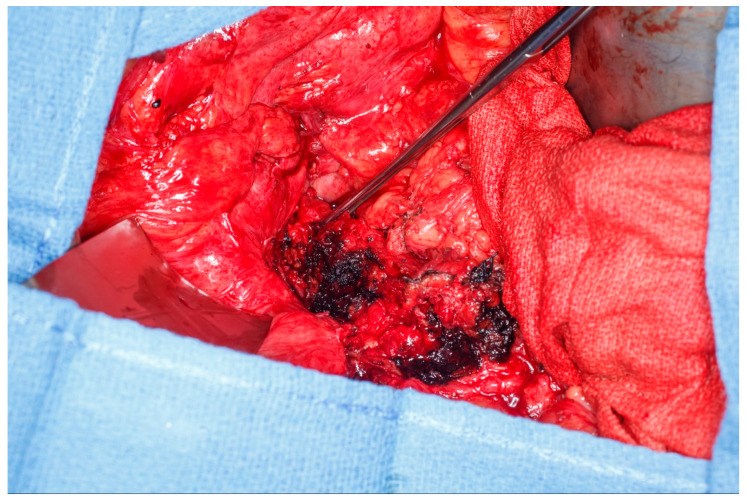
Perivesical fat flap dissected off the posterior bladder and dropped into the pelvis, thus occluding the pelvic inlet and preventing small bowel from falling into the pelvis. (Used with permission from the Mayo Foundation for Medical Education and Research; all rights reserved).

This patient was found to have no remaining greater omentum at the time of surgery. He had a significant ventral abdominal wall hernia from previous surgeries that rendered the rectus abdominus muscle no longer a viable option for a flap. He also had significant complex fistulizing perianal disease extending to his groins, and the plastic surgeon did not want to use gracilis flaps initially due to the substantial risk of infection and flap failure. It was felt that gracilis flaps should be kept for a potentially delayed perineal reconstruction if needed. This patient had a significant layer of posterior perivesical fat that could be safely dissected off the back of the bladder. An attachment to the bladder was maintained inferiorly and helped preserve the flap’s blood supply. This fat pad was then dropped down into the pelvis and occluded the pelvic inlet, thus preventing the small bowel from entering the pelvis (See Figure 4). A single drain was left in the pelvis with the tip below the perivesical fat flap.

### 3.4. Gerota’s Fat Pad Flap

**Figure 5:**
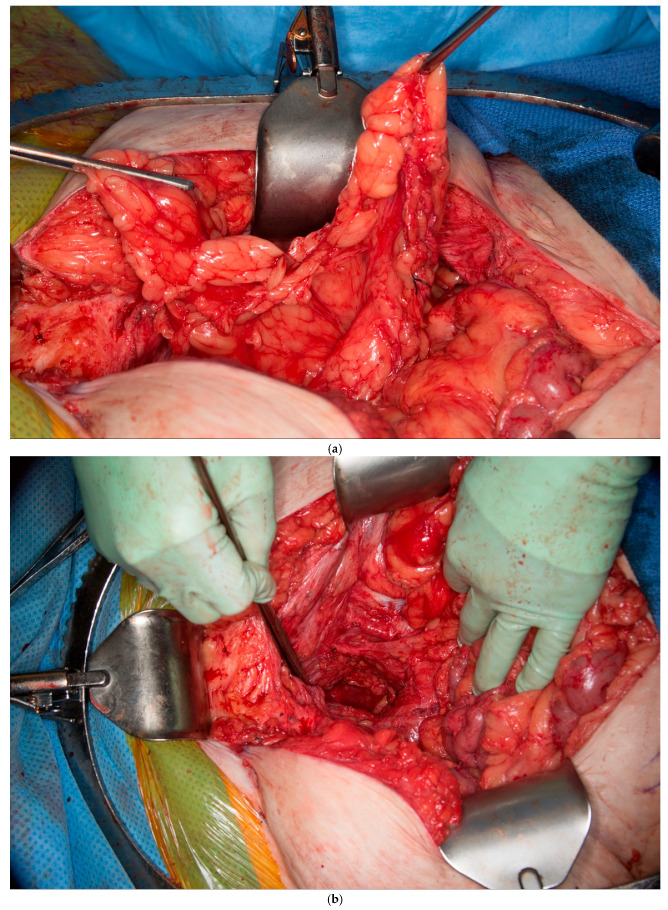

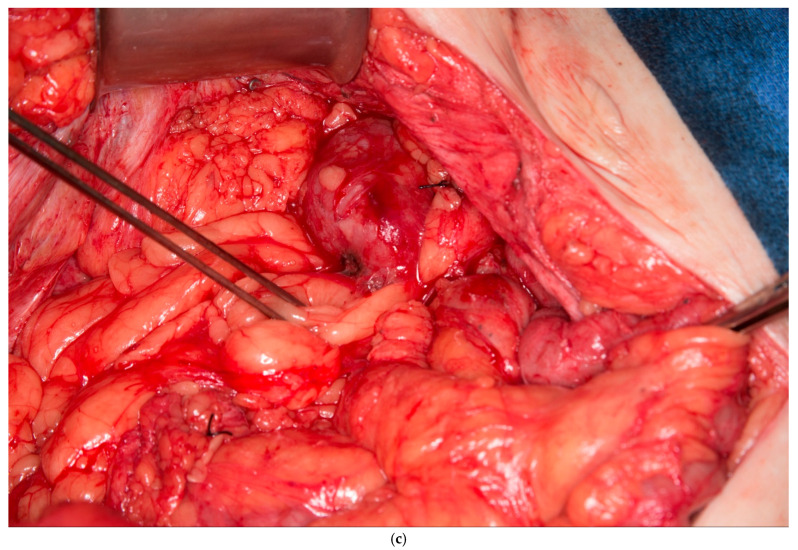
(**a**) Pedicled Gerota’s fat pad flap elevated off the anterior surface of the right kidney. (Used with permission from the Mayo Foundation for Medical Education and Research; all rights reserved). (**b**) Empty pelvis after proctectomy. (Used with permission from the Mayo Foundation for Medical Education and Research; all rights reserved). (**c**) Pelvis filled and pelvic inlet occluded using the pedicled Gerota’s fat pad flap. (Used with permission from the Mayo Foundation for Medical Education and Research; all rights reserved).

This patient was found to have no remaining greater omentum. She had a previous repair of a large ventral hernia with mesh, which rendered the rectus abdominus muscle no longer a viable option for a flap. She required a proctectomy because of multiple complex perianal fistulae despite diversion. While mobilizing the patient’s small intestine of the retroperitoneum, it was recognized that she had a very fatty Gerota’s fat pad overlying her right kidney. This Gerota’s fascia was opened, and the fat pad was peeled off the anterior wall of her kidney (See Figure 5a). With the assistance of a plastic surgeon, a posteromedial attachment of the flap was maintained to ensure adequate blood supply was preserved. Once fully mobilized, the flap easily reached and filled the pelvis (See Figure 5b,c). It was secured in place using 3-0 PDS sutures. A single drain was placed in the pelvis at the end of the case.

### 3.5. Bovine Pericardial Patch-Assisted Pelvic Exclusion

This patient was found to have inadequate remaining omentum to facilitate an OPF. She required a proctectomy because of a rectovaginal fistula. No other possible local pedicled flap options were identified. Once the proctectomy was completed, a 6 cm × 8 cm piece of bovine pericardial patch was used to occlude the pelvic inlet and prevent the bowel from going into the pelvis. It was secured circumferentially using 0-vicryl sutures. Two 19 Fr round channel drains were placed into the pelvis, running laterally to the bovine pericardial patch on each side.

## 4. Discussion

Several well-known short and long-term complications can arise from the dead space associated with an empty pelvis and from the perineal wound in patients who have undergone proctectomy for CD. In the early postoperative phase, sterile and infected pelvic fluid collections can mandate re-admission to the hospital for interventional radiology-guided drainage procedures and result in perineal wound breakdown as fluid tracks through the wound in an attempt to drain. In the intermediate term, many patients suffer from delayed perineal wound healing, chronic wound issues, and the formation of sinus tracts from their perineal skin. Failure of perineal wound healing has been reported in up to 58% of patients at six months from the date of their surgery, and approximately 20% of patients remain unhealed at one year [8,9]. Long-term complications include a small risk of developing a perineal hernia and the risk of developing a small bowel obstruction secondary to loops of small bowel becoming adherent to the proctectomy resection bed in the pelvis [10,11]. Omental pedicle flaps (OPFs) and myofasciocutaneous flaps have traditionally been used to try and mitigate the risk of these complications. However, these options are not always available or suitable [6].

The ability of pelvic flaps to reduce postoperative complications, such as abscess formation and perineal wound breakdown, remains somewhat uncertain. A recent publication from our institution assessing the outcomes for patients who underwent proctectomy for CD, with and without the use of an OPF, failed to identify a difference in outcomes between the two cohorts. The study was somewhat biased in that the patients who had an OPF were more likely to use tobacco, to have a perianal fistula, to have undergone extra sphincteric dissection, and to require a perineal incisional wound VAC, all of which likely equates to a higher risk perineal wound [5]. Limited long-term follow-up data did not allow us to report on perineal hernia rates and re-admissions secondary to small bowel obstruction. There is some data to support the use of OPFs in the setting of abdominoperineal resection for rectal cancer, with those who had flap-assisted closure being afforded improved primary wound healing, shortened duration of healing, reduced wound infection rates, and reduced chronic perineal sinus rates [12,13]. As described in our cohort, CD patients can present unique challenges when trying to deal with the empty pelvis after proctectomy, and circumstances can arise when the greater omentum has been previously resected or is too diminutive to facilitate the formation of an OPF.

Furthermore, there are also circumstances when an immediate reconstruction using a myofasciocutaneous flap is not desirable, such as when the patient has limited reconstruction options, and there is a high risk of failure with reconstruction at the time of proctectomy. In these circumstances, a local pedicled flap using native intra-abdominal tissue to occlude the pelvis can be considered. Of the six patients reported on in this study, there were no prolonged perineal wound healing issues, and only one patient was re-admitted with a pelvic abscess that required drainage. Based on our median follow-up time of 26 months, we have not identified a single perineal hernia or an episode of small bowel obstruction after discharge from the hospital in this cohort. These results are broadly similar to the outcomes experienced in our OPF cohort, although we do acknowledge that the conclusions that can be drawn from a comparison of only six patients are limited. In our opinion, it seems biologically plausible that excluding the small bowel from the pelvis using a local pedicled flap or some other biological material would result in a reduced risk of perineal hernia formation, or small bowel obstruction secondary to a loop of intestine becoming adherent to a raw surface in the pelvis. These local pedicled flaps also have the advantage of not resulting in a second wound or potential loss of function, contrasting with some of the commonly used myofasciocutaneous flaps [14]. Theoretical risks include potential injury to a nearby anatomical structure, such as the bladder in the case of the perivesical fat flap or the kidney in the case of the Gerota’s fat pad flap, flap necrosis, bleeding, and a post-operative collection or abscess that requires radiological guided drainage.

There may also be some merit in placing well-vascularized tissue into the pelvis for perineal wound healing. Previous studies have shown that the omentum has many diverse functions, including neovascularization, hemostasis, tissue healing, regeneration, immune regulation, and acts as an in vivo incubator for specific cell types [15,16,17,18]. Some local tissue-pedicled flaps we described might harbor the same properties as greater omentum. In contrast to this, despite our use of sigmoid mesocolic flaps, there is some evidence limited to a small case series in the published literature that suggests incomplete mesorectal excision in the setting of CD might be associated with impaired wound healing. The mesorectum in CD appears to contain increased numbers of tumor necrosis factor alpha-producing CD14^+^ macrophages and reduced levels of the wound healing marker CD206. Interestingly, re-operating on patients who have undergone close rectal dissection for CD and removing the remaining mesorectum positively affects perineal wound healing [19]. With that in mind, we would recommend careful consideration when deciding whether to use a sigmoid mesocolic flap or not, and they are probably best used in the setting of isolated rectal CD without sigmoid colon or mesocolon involvement.

This study describes several creative techniques to fill the pelvis in patients with CD undergoing proctectomy when omentum is not available, and a myofasciocutaneous flap is not desirable. While there is no direct comparison group to compare this cohort of patients, they appear to have similar outcomes to those described in our omental pedicle flap study. Furthermore, the techniques described are relatively straightforward, can be carried out by a colorectal surgeon with the assistance of a plastic surgeon in some cases, and appear to have a very low risk of complications. We also highlight several simple techniques to ensure adequate perfusion to the flap. The main limitations include the study’s retrospective nature, the small number of patients included, the lack of a comparative cohort, the lack of validation data and the short follow up time. The techniques described are not intended to replace any current standard flap options, but merely as an alternative for patients where limited options exist.

## 5. Conclusions

Several patient factors, including the size of the pelvic inlet, volume of dead space, and distribution of intra-abdominal adipose tissue, should be taken into consideration when attempting to fill the pelvis without the use of an OPF or a myofasciocutaneous flap. Well-vascularized tissue, such as the sigmoid mesocolic flap, may become available secondary to the resection. Mobilizing adjacent tissue from elsewhere in the abdomen may result in an adequate volume of healthy tissue, such as the Gerota’s fat pad flap, the preperitoneal fat and falciform ligament flap, or the perivesical fat flap. Lastly, in exceptional circumstances, non-native material may occlude the pelvic inlet, as demonstrated by the bovine pericardial patch. Local pedicled flaps using native tissue can be added to the armamentarium of any colon and rectal surgeon offering proctectomy for Crohn’s disease.

## Figures and Tables

**Table 1:** Clinical and operative variables for patients included in the study.

Clinical Variable	Patient 1	Patient 2	Patient 3	Patient 4	Patient 5	Patient 6
**Gender**	Female	Female	Female	Male	Female	Male
**Age (years)**	50	73	42	32	59	75
**Surgical indication**	Fistulizing perianal disease	Rectovaginal fistula	Rectovaginal fistula and anal stenosis	Fistulizing perianal disease	Fistulizing perianal disease and rectovaginal fistula	Dysplasia
**Operation**	Completion proctectomy	Proctectomy	Completion proctectomy	Proctectomy and small bowel resection	Total proctocolectomy	Completion proctocolectomy
**Type of flap**	Gerota’s fat pad	Bovine pericardial patch	Peritoneum/preperitoneal fat/falciform ligament	Perivesical fat	Sigmoid mesocolon	Sigmoid mesocolon and posterior bladder peritoneum
**Diabetes**	No	No	No	No	No	No
**BMI (kg/m^2^)**	52.0	18.1	31.1	22.9	30.3	25.4
**Immunosuppressants**	No	No	Ustekinumab	No	Ustekinumab	No
**Perianal fistula**	Yes	Yes	Yes	Yes	Yes	No
**Perineal dissection**	Intersphincteric	Mucosectomy	Intersphincteric	Intersphincteric	Extrasphincteric	Intersphincteric
**Perineal skin closure**	2-0 Nylon	3-0 Monocryl	2-0 Nylon	2-0 Nylon	2-0 Nylon	2-0 Nylon
**Operative time (mins)**	296	271	479	585	463	509
**Blood loss (mL)**	500	200	1860	750	500	200
**Length of stay (days)**	3	13	5	5	17	4
**Re-admission**	No	No	No	Yes	No	No
**Return to the OR**	No	No	No	No	No	No
**Need for postop IR guided pelvic drain**	No	No	No	Yes	No	No
**Perineal wound issues**	No	No	No	No	No	No

**Table 2:** Pathological variables for patients included in the study.

Pathological Variable	Patient 1	Patient 2	Patient 3	Patient 4	Patient 5	Patient 6
**Sharply demarcated segment**	No	No	No	No	Yes	No
**Linear ulceration**	No	Yes	No	No	Yes	No
**Skip areas**	No	Yes	No	No	Yes	No
**Continuous involvement**	No	No	No	No	No	No
**Fat creeping**	No	No	Yes	Yes	Yes	Yes
**Fissure**	No	No	No	Yes	Yes	No
**Fistula**	No	No	No	No	No	No
**Stricture**	Yes	Yes	No	Yes	Yes	No
**Marked mucosal atrophy**	No	No	No	No	No	No
**Transmural inflammation**	No	Yes	No	Yes	Yes	No
**Fissuring ulceration**	No	Yes	No	Yes	No	Yes
**Non necrotizing granulomata**	No	No	No	No	No	No
**Lymphoid aggregates**	No	Yes	No	No	Yes	No
**Dense mucosal inflammation**	No	No	No	Yes	No	No
**Crypt abscesses**	No	No	No	No	No	No
**Thickening of muscularis mucosa**	No	No	No	No	No	Yes
**Proximal margin involvement**	No	No	No	No	Yes	No
**Distal margin involvement**	No	No	No	No	No	No

## Data Availability

Original data is available upon reasonable request from the authors.
